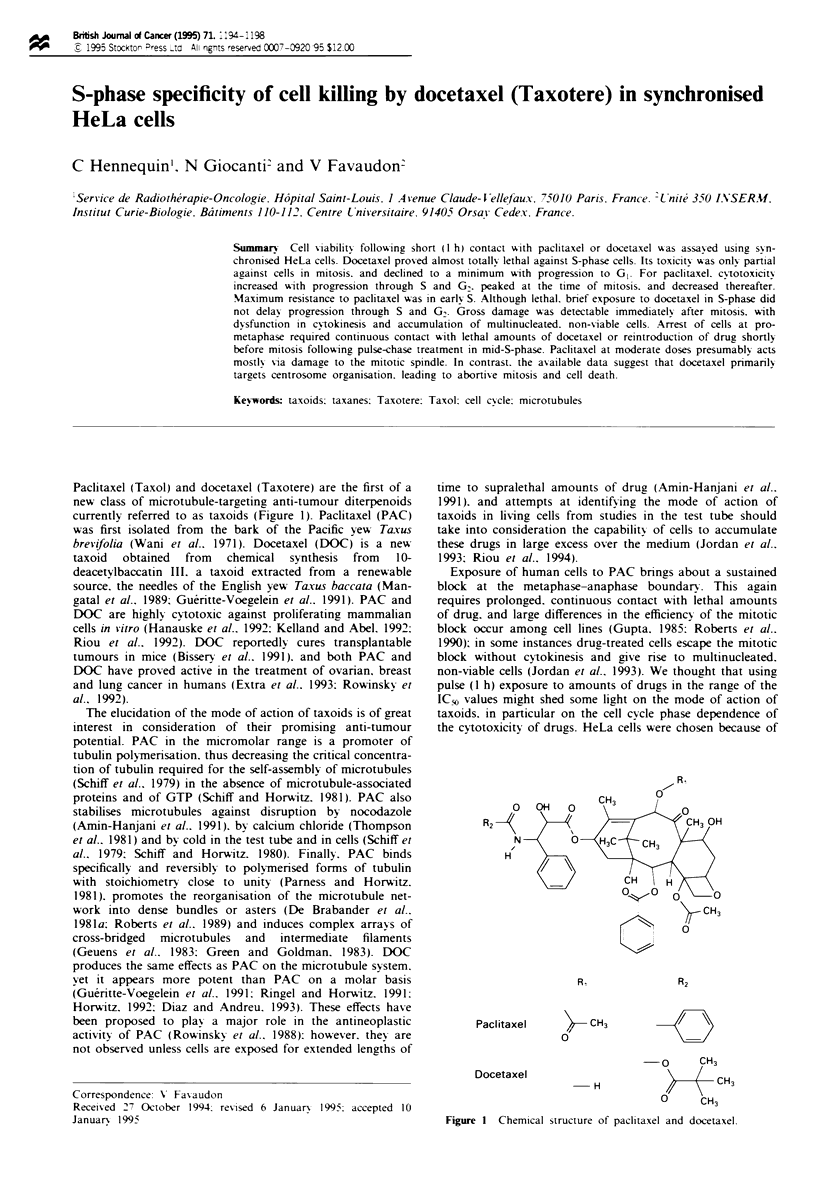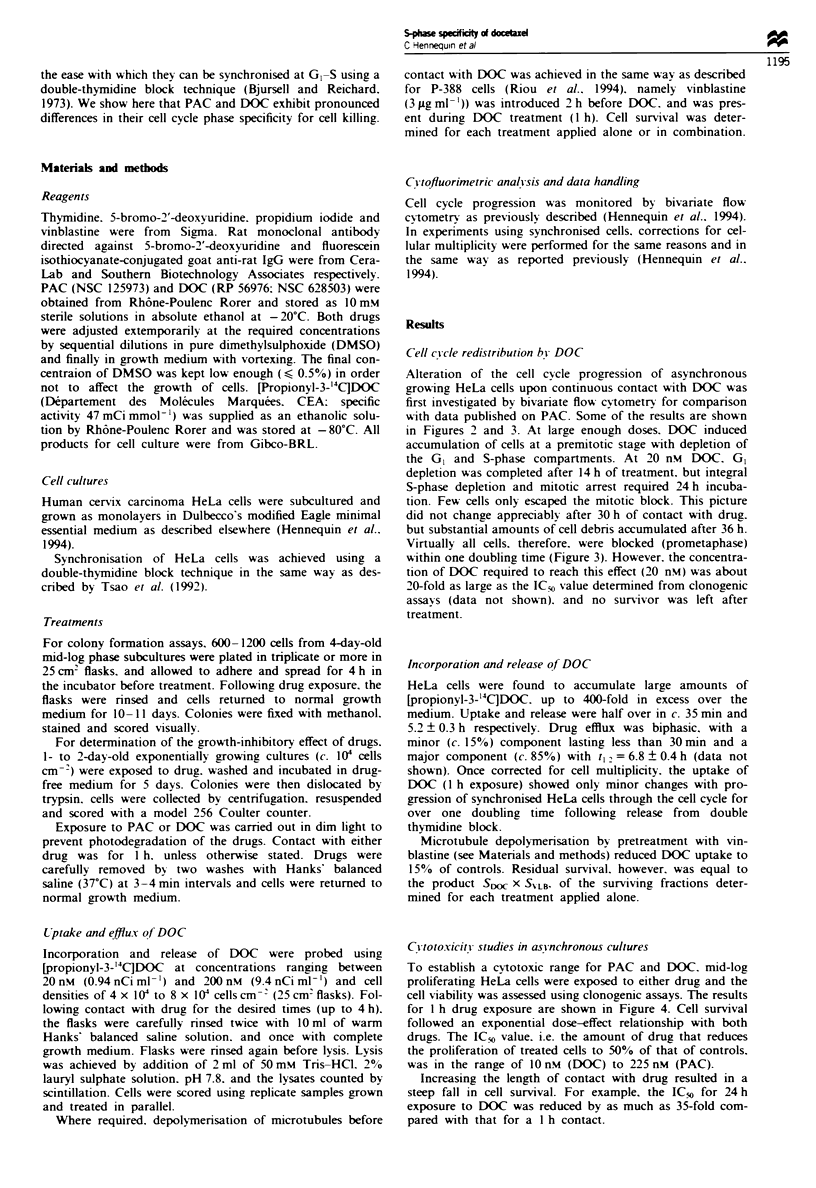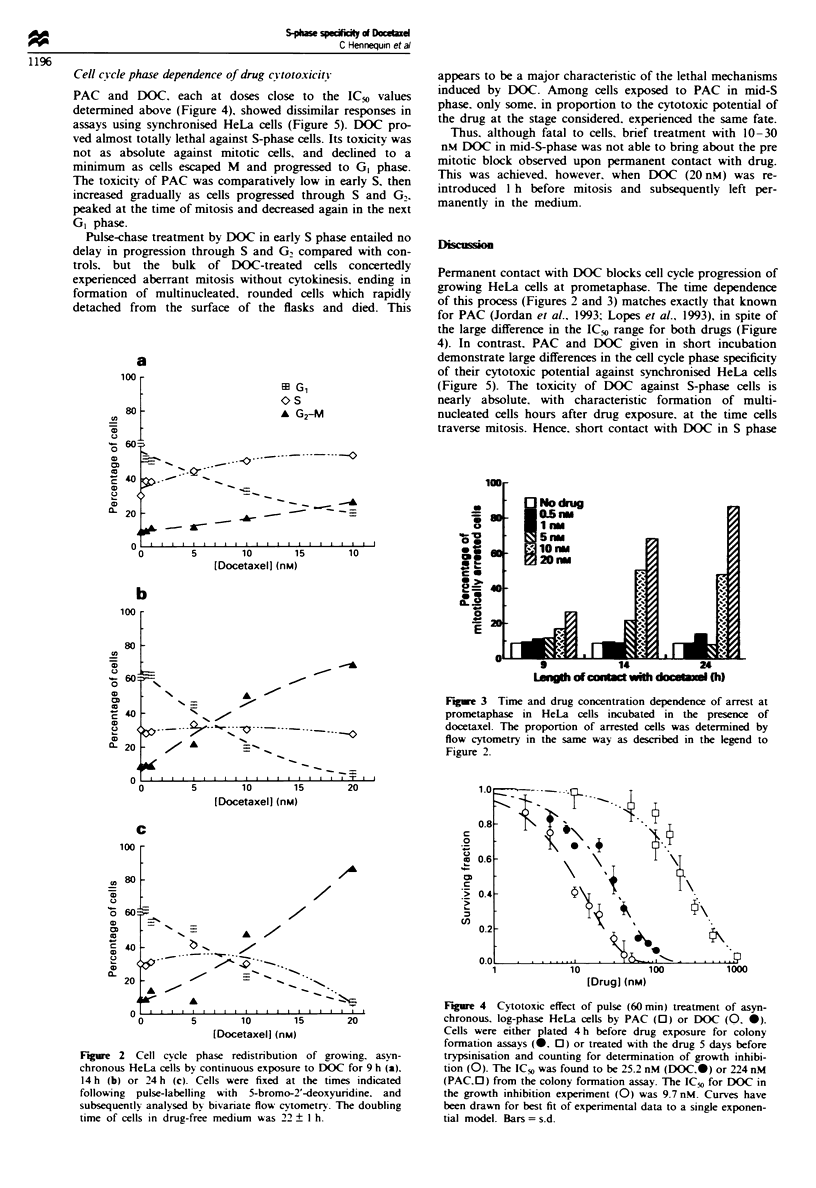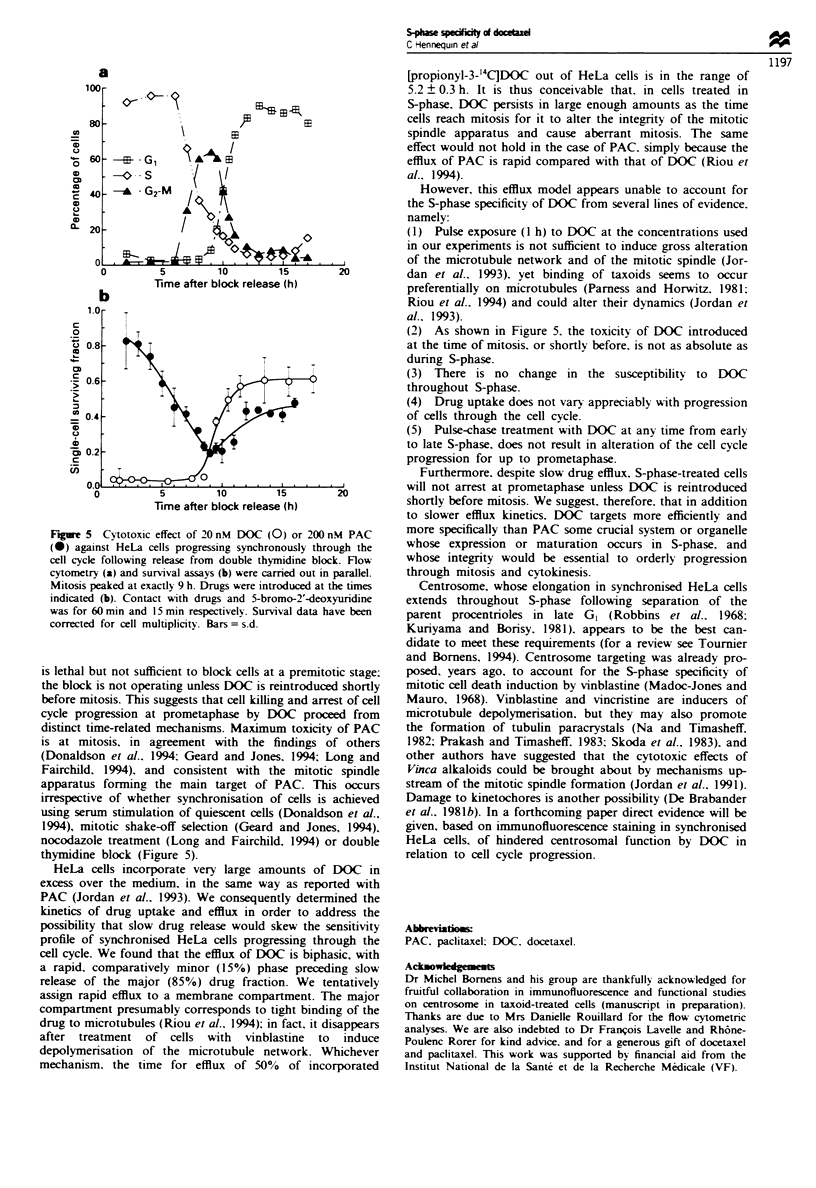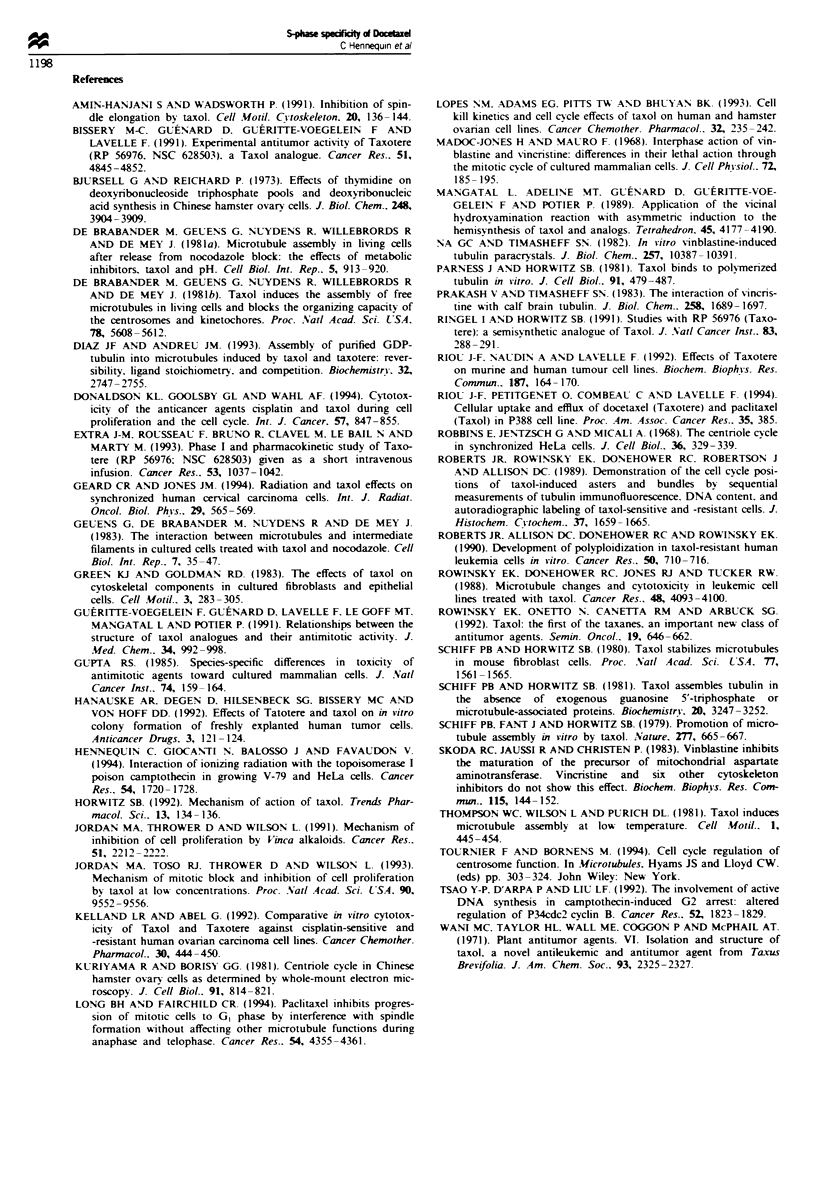# S-phase specificity of cell killing by docetaxel (Taxotere) in synchronised HeLa cells.

**DOI:** 10.1038/bjc.1995.232

**Published:** 1995-06

**Authors:** C. Hennequin, N. Giocanti, V. Favaudon

**Affiliations:** Service de Radiothérapie-Oncologie, Hôpital Saint-Louis, Paris, France.

## Abstract

Cell viability following short (1 h) contact with paclitaxel or docetaxel was assayed using synchronised HeLa cells. Docetaxel proved almost totally lethal against S-phase cells. Its toxicity was only partial against cells in mitosis, and declined to a minimum with progression to G1. For paclitaxel, cytotoxicity increased with progression through S and G2, peaked at the time of mitosis, and decreased thereafter. Maximum resistance to paclitaxel was in early S. Although lethal, brief exposure to docetaxel in S-phase did not delay progression through S and G2. Gross damage was detectable immediately after mitosis, with dysfunction in cytokinesis and accumulation of multinucleated, non-viable cells. Arrest of cells at prometaphase required continuous contact with lethal amounts of docetaxel or reintroduction of drug shortly before mitosis following pulse-chase treatment in mid-S-phase. Paclitaxel at moderate doses presumably acts mostly via damage to the mitotic spindle. In contrast, the available data suggest that docetaxel primarily targets centrosome organisation, leading to abortive mitosis and cell death.


British Journal of Cancer (1995) 71, 1194–1198

© 1995 Stockton Press Ltd All rights reserved 0007–0920/95 $12.00

### S-phase specificity of cell killing by docetaxel (Taxotere) in synchronised HeLa cells

C Hennequin[1], N Giocanti[2] and V Favaudon[2]

[1]Service de Radiothérapie-Oncologie, Hôpital Saint-Louis, 1 Avenue Claude-Vellefaux, 75010 Paris, France; [2]Unité 350 INSERM, Institut Curie-Biologie, Bâtiments 110-112, Centre Universitaire, 91405 Orsay Cedex, France.

**Summary** Cell viability following short (1 h) contact with paclitaxel or docetaxel was assayed using synchronised HeLa cells. Docetaxel proved almost totally lethal against S-phase cells. Its toxicity was only partial against cells in mitosis, and declined to a minimum with progression to $G_1$. For paclitaxel, cytotoxicity increased with progression through S and $G_2$, peaked at the time of mitosis, and decreased thereafter. Maximum resistance to paclitaxel was in early S. Although lethal, brief exposure to docetaxel in S-phase did not delay progression through S and $G_2$. Gross damage was detectable immediately after mitosis, with dysfunction in cytokinesis and accumulation of multinucleated, non-viable cells. Arrest of cells at prometaphase required continuous contact with lethal amounts of docetaxel or reintroduction of drug shortly before mitosis following pulse-chase treatment in mid-S-phase. Paclitaxel at moderate doses presumably acts mostly via damage to the mitotic spindle. In contrast, the available data suggest that docetaxel primarily targets centrosome organisation, leading to abortive mitosis and cell death.

**Keywords:** taxoids; taxanes; Taxotere; Taxol; cell cycle; microtubules

Paclitaxel (Taxol) and docetaxel (Taxotere) are the first of a new class of microtubule-targeting anti-tumour diterpenoids currently referred to as taxoids (Figure 1). Paclitaxel (PAC) was first isolated from the bark of the Pacific yew *Taxus brevifolia* (Wani *et al*., 1971). Docetaxel (DOC) is a new taxoid obtained from chemical synthesis from 10-deacetylbaccatin III, a taxoid extracted from a renewable source, the needles of the English yew *Taxus baccata* (Mangatal *et al*., 1989; Guéritte-Voegelein *et al*., 1991). PAC and DOC are highly cytotoxic against proliferating mammalian cells *in vitro* (Hanauske *et al*., 1992; Kelland and Abel, 1992; Riou *et al*., 1992). DOC reportedly cures transplantable tumours in mice (Bissery *et al*., 1991), and both PAC and DOC have proved active in the treatment of ovarian, breast and lung cancer in humans (Extra *et al*., 1993; Rowinsky *et al*., 1992).

The elucidation of the mode of action of taxoids is of great interest in consideration of their promising anti-tumour potential. PAC in the micromolar range is a promoter of tubulin polymerisation, thus decreasing the critical concentration of tubulin required for the self-assembly of microtubules (Schiff *et al*., 1979) in the absence of microtubule-associated proteins and of GTP (Schiff and Horwitz, 1981). PAC also stabilises microtubules against disruption by nocodazole (Amin-Hanjani *et al*., 1991), by calcium chloride (Thompson *et al*., 1981) and by cold in the test tube and in cells (Schiff *et al*., 1979; Schiff and Horwitz, 1980). Finally, PAC binds specifically and reversibly to polymerised forms of tubulin with stoichiometry close to unity (Parness and Horwitz, 1981), promotes the reorganisation of the microtubule network into dense bundles or asters (De Brabander *et al*., 1981*a*; Roberts *et al*., 1989) and induces complex arrays of cross-bridged microtubules and intermediate filaments (Geuens *et al*., 1983; Green and Goldman, 1983). DOC produces the same effects as PAC on the microtubule system, yet it appears more potent than PAC on a molar basis (Guéritte-Voegelein *et al*., 1991; Ringel and Horwitz, 1991; Horwitz, 1992; Diaz and Andreu, 1993). These effects have been proposed to play a major role in the antineoplastic activity of PAC (Rowinsky *et al*., 1988); however, they are not observed unless cells are exposed for extended lengths of time to supralethal amounts of drug (Amin-Hanjani *et al*., 1991), and attempts at identifying the mode of action of taxoids in living cells from studies in the test tube should take into consideration the capability of cells to accumulate these drugs in large excess over the medium (Jordan *et al*., 1993; Riou *et al*., 1994).

Exposure of human cells to PAC brings about a sustained block at the metaphase–anaphase boundary. This again requires prolonged, continuous contact with lethal amounts of drug, and large differences in the efficiency of the mitotic block occur among cell lines (Gupta, 1985; Roberts *et al*., 1990); in some instances drug-treated cells escape the mitotic block without cytokinesis and give rise to multinucleated, non-viable cells (Jordan *et al*., 1993). We thought that using pulse (1 h) exposure to amounts of drugs in the range of the $IC_{50}$ values might shed some light on the mode of action of taxoids, in particular on the cell cycle phase dependence of the cytotoxicity of drugs. HeLa cells were chosen because of

**Figure 1** Chemical structure of paclitaxel and docetaxel.

Correspondence: V Favaudon
Received 27 October 1994; revised 6 January 1995; accepted 10 January 1995

the ease with which they can be synchronised at $G_1$–S using a double-thymidine block technique (Bjursell and Reichard, 1973). We show here that PAC and DOC exhibit pronounced differences in their cell cycle phase specificity for cell killing.

### Materials and methods

*Reagents*

Thymidine, 5-bromo-2'-deoxyuridine, propidium iodide and vinblastine were from Sigma. Rat monoclonal antibody directed against 5-bromo-2'-deoxyuridine and fluorescein isothiocyanate-conjugated goat anti-rat IgG were from Cera-Lab and Southern Biotechnology Associates respectively. PAC (NSC 125973) and DOC (RP 56976; NSC 628503) were obtained from Rhône-Poulenc Rorer and stored as 10 mM sterile solutions in absolute ethanol at −20°C. Both drugs were adjusted extemporarily at the required concentrations by sequential dilutions in pure dimethylsulphoxide (DMSO) and finally in growth medium with vortexing. The final concentraion of DMSO was kept low enough ($\leqslant$ 0.5%) in order not to affect the growth of cells. [Propionyl-3-$^{14}$C]DOC (Département des Molécules Marquées, CEA; specific activity 47 mCi mmol$^{-1}$) was supplied as an ethanolic solution by Rhône-Poulenc Rorer and was stored at −80°C. All products for cell culture were from Gibco-BRL.

*Cell cultures*

Human cervix carcinoma HeLa cells were subcultured and grown as monolayers in Dulbecco's modified Eagle minimal essential medium as described elsewhere (Hennequin *et al*., 1994).

Synchronisation of HeLa cells was achieved using a double-thymidine block technique in the same way as described by Tsao *et al*. (1992).

*Treatments*

For colony formation assays, 600–1200 cells from 4-day-old mid-log phase subcultures were plated in triplicate or more in 25 cm$^2$ flasks, and allowed to adhere and spread for 4 h in the incubator before treatment. Following drug exposure, the flasks were rinsed and cells returned to normal growth medium for 10–11 days. Colonies were fixed with methanol, stained and scored visually.

For determination of the growth-inhibitory effect of drugs, 1- to 2-day-old exponentially growing cultures (*c*. $10^4$ cells cm$^{-2}$) were exposed to drug, washed and incubated in drug-free medium for 5 days. Colonies were then dislocated by trypsin, cells were collected by centrifugation, resuspended and scored with a model 256 Coulter counter.

Exposure to PAC or DOC was carried out in dim light to prevent photodegradation of the drugs. Contact with either drug was for 1 h, unless otherwise stated. Drugs were carefully removed by two washes with Hanks' balanced saline (37°C) at 3–4 min intervals and cells were returned to normal growth medium.

*Uptake and efflux of DOC*

Incorporation and release of DOC were probed using [propionyl-3-$^{14}$C]DOC at concentrations ranging between 20 nM (0.94 nCi ml$^{-1}$) and 200 nM (9.4 nCi ml$^{-1}$) and cell densities of $4 \times 10^4$ to $8 \times 10^4$ cells cm$^{-2}$ (25 cm$^2$ flasks). Following contact with drug for the desired times (up to 4 h), the flasks were carefully rinsed twice with 10 ml of warm Hanks' balanced saline solution, and once with complete growth medium. Flasks were rinsed again before lysis. Lysis was achieved by addition of 2 ml of 50 mM Tris–HCl, 2% lauryl sulphate solution, pH 7.8, and the lysates counted by scintillation. Cells were scored using replicate samples grown and treated in parallel.

Where required, depolymerisation of microtubules before contact with DOC was achieved in the same way as described for P-388 cells (Riou *et al*., 1994), namely vinblastine (3 µg ml$^{-1}$)) was introduced 2 h before DOC, and was present during DOC treatment (1 h). Cell survival was determined for each treatment applied alone or in combination.

*Cytofluorimetric analysis and data handling*

Cell cycle progression was monitored by bivariate flow cytometry as previously described (Hennequin *et al*., 1994). In experiments using synchronised cells, corrections for cellular multiplicity were performed for the same reasons and in the same way as reported previously (Hennequin *et al*., 1994).

### Results

*Cell cycle redistribution by DOC*

Alteration of the cell cycle progression of asynchronous growing HeLa cells upon continuous contact with DOC was first investigated by bivariate flow cytometry for comparison with data published on PAC. Some of the results are shown in Figures 2 and 3. At large enough doses, DOC induced accumulation of cells at a premitotic stage with depletion of the $G_1$ and S-phase compartments. At 20 nM DOC, $G_1$ depletion was completed after 14 h of treatment, but integral S-phase depletion and mitotic arrest required 24 h incubation. Few cells only escaped the mitotic block. This picture did not change appreciably after 30 h of contact with drug, but substantial amounts of cell debris accumulated after 36 h. Virtually all cells, therefore, were blocked (prometaphase) within one doubling time (Figure 3). However, the concentration of DOC required to reach this effect (20 nM) was about 20-fold as large as the $IC_{50}$ value determined from clonogenic assays (data not shown), and no survivor was left after treatment.

*Incorporation and release of DOC*

HeLa cells were found to accumulate large amounts of [propionyl-3-$^{14}$C]DOC, up to 400-fold in excess over the medium. Uptake and release were half over in *c*. 35 min and 5.2 ± 0.3 h respectively. Drug efflux was biphasic, with a minor (*c*. 15%) component lasting less than 30 min and a major component (*c*. 85%) with $t_{1/2}$ = 6.8 ± 0.4 h (data not shown). Once corrected for cell multiplicity, the uptake of DOC (1 h exposure) showed only minor changes with progression of synchronised HeLa cells through the cell cycle for over one doubling time following release from double thymidine block.

Microtubule depolymerisation by pretreatment with vinblastine (see Materials and methods) reduced DOC uptake to 15% of controls. Residual survival, however, was equal to the product $S_{DOC} \times S_{VLB}$, of the surviving fractions determined for each treatment applied alone.

*Cytotoxicity studies in asynchronous cultures*

To establish a cytotoxic range for PAC and DOC, mid-log proliferating HeLa cells were exposed to either drug and the cell viability was assessed using clonogenic assays. The results for 1 h drug exposure are shown in Figure 4. Cell survival followed an exponential dose–effect relationship with both drugs. The $IC_{50}$ value, i.e. the amount of drug that reduces the proliferation of treated cells to 50% of that of controls, was in the range of 10 nM (DOC) to 225 nM (PAC).

Increasing the length of contact with drug resulted in a steep fall in cell survival. For example, the $IC_{50}$ for 24 h exposure to DOC was reduced by as much as 35-fold compared with that for a 1 h contact.

*Cell cycle phase dependence of drug cytotoxicity*

PAC and DOC, each at doses close to the $IC_{50}$ values determined above (Figure 4), showed dissimilar responses in assays using synchronised HeLa cells (Figure 5). DOC proved almost totally lethal against S-phase cells. Its toxicity was not as absolute against mitotic cells, and declined to a minimum as cells escaped M and progressed to $G_1$ phase. The toxicity of PAC was comparatively low in early S, then increased gradually as cells progressed through S and $G_2$, peaked at the time of mitosis and decreased again in the next $G_1$ phase.

Pulse-chase treatment by DOC in early S phase entailed no delay in progression through S and $G_2$ compared with controls, but the bulk of DOC-treated cells concertedly experienced aberrant mitosis without cytokinesis, ending in formation of multinucleated, rounded cells which rapidly detached from the surface of the flasks and died. This appears to be a major characteristic of the lethal mechanisms induced by DOC. Among cells exposed to PAC in mid-S phase, only some, in proportion to the cytotoxic potential of the drug at the stage considered, experienced the same fate.

Thus, although fatal to cells, brief treatment with 10–30 nM DOC in mid-S-phase was not able to bring about the pre mitotic block observed upon permanent contact with drug. This was achieved, however, when DOC (20 nM) was reintroduced 1 h before mitosis and subsequently left permanently in the medium.

### Discussion

Permanent contact with DOC blocks cell cycle progression of growing HeLa cells at prometaphase. The time dependence of this process (Figures 2 and 3) matches exactly that known for PAC (Jordan *et al*., 1993; Lopes *et al*., 1993), in spite of the large difference in the $IC_{50}$ range for both drugs (Figure 4). In contrast, PAC and DOC given in short incubation demonstrate large differences in the cell cycle phase specificity of their cytotoxic potential against synchronised HeLa cells (Figure 5). The toxicity of DOC against S-phase cells is nearly absolute, with characteristic formation of multinucleated cells hours after drug exposure, at the time cells traverse mitosis. Hence, short contact with DOC in S phase

**Figure 2** Cell cycle phase redistribution of growing, asynchronous HeLa cells by continuous exposure to DOC for 9 h (**a**), 14 h (**b**) or 24 h (**c**). Cells were fixed at the times indicated following pulse-labelling with 5-bromo-2'-deoxyuridine, and subsequently analysed by bivariate flow cytometry. The doubling time of cells in drug-free medium was 22 ± 1 h.

**Figure 3** Time and drug concentration dependence of arrest at prometaphase in HeLa cells incubated in the presence of docetaxel. The proportion of arrested cells was determined by flow cytometry in the same way as described in the legend to Figure 2.

**Figure 4** Cytotoxic effect of pulse (60 min) treatment of asynchronous, log-phase HeLa cells by PAC (□) or DOC (○, ●). Cells were either plated 4 h before drug exposure for colony formation assays (●, □) or treated with the drug 5 days before trypsinisation and counting for determination of growth inhibition (○). The $IC_{50}$ was found to be 25.2 nM (DOC,●) or 224 nM (PAC,□) from the colony formation assay. The $IC_{50}$ for DOC in the growth inhibition experiment (○) was 9.7 nM. Curves have been drawn for best fit of experimental data to a single exponential model. Bars = s.d.

**Figure 5** Cytotoxic effect of 20 nM DOC (○) or 200 nM PAC (●) against HeLa cells progressing synchronously through the cell cycle following release from double thymidine block. Flow cytometry (**a**) and survival assays (**b**) were carried out in parallel. Mitosis peaked at exactly 9 h. Drugs were introduced at the times indicated (**b**). Contact with drugs and 5-bromo-2′-deoxyuridine was for 60 min and 15 min respectively. Survival data have been corrected for cell multiplicity. Bars = s.d.

is lethal but not sufficient to block cells at a premitotic stage: the block is not operating unless DOC is reintroduced shortly before mitosis. This suggests that cell killing and arrest of cell cycle progression at prometaphase by DOC proceed from distinct time-related mechanisms. Maximum toxicity of PAC is at mitosis, in agreement with the findings of others (Donaldson *et al.*, 1994; Geard and Jones, 1994; Long and Fairchild, 1994), and consistent with the mitotic spindle apparatus forming the main target of PAC. This occurs irrespective of whether synchronisation of cells is achieved using serum stimulation of quiescent cells (Donaldson *et al.*, 1994), mitotic shake-off selection (Geard and Jones, 1994), nocodazole treatment (Long and Fairchild, 1994) or double thymidine block (Figure 5).

HeLa cells incorporate very large amounts of DOC in excess over the medium, in the same way as reported with PAC (Jordan *et al.*, 1993). We consequently determined the kinetics of drug uptake and efflux in order to address the possibility that slow drug release would skew the sensitivity profile of synchronised HeLa cells progressing through the cell cycle. We found that the efflux of DOC is biphasic, with a rapid, comparatively minor (15%) phase preceding slow release of the major (85%) drug fraction. We tentatively assign rapid efflux to a membrane compartment. The major compartment presumably corresponds to tight binding of the drug to microtubules (Riou *et al.*, 1994); in fact, it disappears after treatment of cells with vinblastine to induce depolymerisation of the microtubule network. Whichever mechanism, the time for efflux of 50% of incorporated [propionyl-3-$^{14}$C]DOC out of HeLa cells is in the range of $5.2 \pm 0.3$ h. It is thus conceivable that, in cells treated in S-phase, DOC persists in large enough amounts as the time cells reach mitosis for it to alter the integrity of the mitotic spindle apparatus and cause aberrant mitosis. The same effect would not hold in the case of PAC, simply because the efflux of PAC is rapid compared with that of DOC (Riou *et al.*, 1994).

However, this efflux model appears unable to account for the S-phase specificity of DOC from several lines of evidence, namely:

(1) Pulse exposure (1 h) to DOC at the concentrations used in our experiments is not sufficient to induce gross alteration of the microtubule network and of the mitotic spindle (Jordan *et al.*, 1993), yet binding of taxoids seems to occur preferentially on microtubules (Parness and Horwitz, 1981; Riou *et al.*, 1994) and could alter their dynamics (Jordan *et al.*, 1993).

(2) As shown in Figure 5, the toxicity of DOC introduced at the time of mitosis, or shortly before, is not as absolute as during S-phase.

(3) There is no change in the susceptibility to DOC throughout S-phase.

(4) Drug uptake does not vary appreciably with progression of cells through the cell cycle.

(5) Pulse-chase treatment with DOC at any time from early to late S-phase, does not result in alteration of the cell cycle progression for up to prometaphase.

Furthermore, despite slow drug efflux, S-phase-treated cells will not arrest at prometaphase unless DOC is reintroduced shortly before mitosis. We suggest, therefore, that in addition to slower efflux kinetics, DOC targets more efficiently and more specifically than PAC some crucial system or organelle whose expression or maturation occurs in S-phase, and whose integrity would be essential to orderly progression through mitosis and cytokinesis.

Centrosome, whose elongation in synchronised HeLa cells extends throughout S-phase following separation of the parent procentrioles in late $G_1$ (Robbins *et al.*, 1968; Kuriyama and Borisy, 1981), appears to be the best candidate to meet these requirements (for a review see Tournier and Bornens, 1994). Centrosome targeting was already proposed, years ago, to account for the S-phase specificity of mitotic cell death induction by vinblastine (Madoc-Jones and Mauro, 1968). Vinblastine and vincristine are inducers of microtubule depolymerisation, but they may also promote the formation of tubulin paracrystals (Na and Timasheff, 1982; Prakash and Timasheff, 1983; Skoda *et al.*, 1983), and other authors have suggested that the cytotoxic effects of *Vinca* alkaloids could be brought about by mechanisms upstream of the mitotic spindle formation (Jordan *et al.*, 1991). Damage to kinetochores is another possibility (De Brabander *et al.*, 1981*b*). In a forthcoming paper direct evidence will be given, based on immunofluorescence staining in synchronised HeLa cells, of hindered centrosomal function by DOC in relation to cell cycle progression.

**Abbreviations:**
PAC, paclitaxel; DOC, docetaxel.

**Acknowledgements**
Dr Michel Bornens and his group are thankfully acknowledged for fruitful collaboration in immunofluorescence and functional studies on centrosome in taxoid-treated cells (manuscript in preparation). Thanks are due to Mrs Danielle Rouillard for the flow cytometric analyses. We are also indebted to Dr François Lavelle and Rhône-Poulenc Rorer for kind advice, and for a generous gift of docetaxel and paclitaxel. This work was supported by financial aid from the Institut National de la Santé et de la Recherche Médicale (VF).

### References

AMIN-HANJANI S AND WADSWORTH P. (1991). Inhibition of spindle elongation by taxol. *Cell Motil. Cytoskeleton*, **20**, 136-144.

BISSERY M-C, GUÉNARD D, GUÉRITTE-VOEGELEIN F AND LAVELLE F. (1991). Experimental antitumor activity of Taxotere (RP 56976, NSC 628503), a Taxol analogue. *Cancer Res.*, **51**, 4845-4852.

BJURSELL G AND REICHARD P. (1973). Effects of thymidine on deoxyribonucleoside triphosphate pools and deoxyribonucleic acid synthesis in Chinese hamster ovary cells. *J. Biol. Chem.*, **248**, 3904-3909.

DE BRABANDER M, GEUENS G, NUYDENS R, WILLEBRORDS R AND DE MEY J. (1981*a*). Microtubule assembly in living cells after release from nocodazole block: the effects of metabolic inhibitors, taxol and pH. *Cell Biol. Int. Rep.*, **5**, 913-920.

DE BRABANDER M, GEUENS G, NUYDENS R, WILLEBRORDS R AND DE MEY J. (1981*b*). Taxol induces the assembly of free microtubules in living cells and blocks the organizing capacity of the centrosomes and kinetochores. *Proc. Natl Acad. Sci. USA*, **78**, 5608-5612.

DIAZ JF AND ANDREU JM. (1993). Assembly of purified GDP-tubulin into microtubules induced by taxol and taxotere: reversibility, ligand stoichiometry, and competition. *Biochemistry*, **32**, 2747-2755.

DONALDSON KL, GOOLSBY GL AND WAHL AF. (1994). Cytotoxicity of the anticancer agents cisplatin and taxol during cell proliferation and the cell cycle. *Int. J. Cancer*, **57**, 847-855.

EXTRA J-M, ROUSSEAU F, BRUNO R, CLAVEL M, LE BAIL N AND MARTY M. (1993). Phase I and pharmacokinetic study of Taxotere (RP 56976; NSC 628503) given as a short intravenous infusion. *Cancer Res.*, **53**, 1037-1042.

GEARD CR AND JONES JM. (1994). Radiation and taxol effects on synchronized human cervical carcinoma cells. *Int. J. Radiat. Oncol. Biol. Phys.*, **29**, 565-569.

GEUENS G, DE BRABANDER M, NUYDENS R AND DE MEY J. (1983). The interaction between microtubules and intermediate filaments in cultured cells treated with taxol and nocodazole. *Cell Biol. Int. Rep.*, **7**, 35-47.

GREEN KJ AND GOLDMAN RD. (1983). The effects of taxol on cytoskeletal components in cultured fibroblasts and epithelial cells. *Cell Motil.*, **3**, 283-305.

GUÉRITTE-VOEGELEIN F, GUÉNARD D, LAVELLE F, LE GOFF MT, MANGATAL L AND POTIER P. (1991). Relationships between the structure of taxol analogues and their antimitotic activity. *J. Med. Chem.*, **34**, 992-998.

GUPTA RS. (1985). Species-specific differences in toxicity of antimitotic agents toward cultured mammalian cells. *J. Natl Cancer Inst.*, **74**, 159-164.

HANAUSKE AR, DEGEN D, HILSENBECK SG, BISSERY MC AND VON HOFF DD. (1992). Effects of Tatotere and taxol on *in vitro* colony formation of freshly explanted human tumor cells. *Anticancer Drugs*, **3**, 121-124.

HENNEQUIN C, GIOCANTI N, BALOSSO J AND FAVAUDON V. (1994). Interaction of ionizing radiation with the topoisomerase I poison camptothecin in growing V-79 and HeLa cells. *Cancer Res.*, **54**, 1720-1728.

HORWITZ SB. (1992). Mechanism of action of taxol. *Trends Pharmacol. Sci.*, **13**, 134-136.

JORDAN MA, THROWER D AND WILSON L. (1991). Mechanism of inhibition of cell proliferation by *Vinca* alkaloids. *Cancer Res.*, **51**, 2212-2222.

JORDAN MA, TOSO RJ, THROWER D AND WILSON L. (1993). Mechanism of mitotic block and inhibition of cell proliferation by taxol at low concentrations. *Proc. Natl Acad. Sci. USA*, **90**, 9552-9556.

KELLAND LR AND ABEL G. (1992). Comparative *in vitro* cytotoxicity of Taxol and Taxotere against cisplatin-sensitive and -resistant human ovarian carcinoma cell lines. *Cancer Chemother. Pharmacol.*, **30**, 444-450.

KURIYAMA R AND BORISY GG. (1981). Centriole cycle in Chinese hamster ovary cells as determined by whole-mount electron microscopy. *J. Cell Biol.*, **91**, 814-821.

LONG BH AND FAIRCHILD CR. (1994). Paclitaxel inhibits progression of mitotic cells to $G_1$ phase by interference with spindle formation without affecting other microtubule functions during anaphase and telophase. *Cancer Res.*, **54**, 4355-4361.

LOPES NM, ADAMS EG, PITTS TW AND BHUYAN BK. (1993). Cell kill kinetics and cell cycle effects of taxol on human and hamster ovarian cell lines. *Cancer Chemother. Pharmacol.*, **32**, 235-242.

MADOC-JONES H AND MAURO F. (1968). Interphase action of vinblastine and vincristine: differences in their lethal action through the mitotic cycle of cultured mammalian cells. *J. Cell Physiol.*, **72**, 185-195.

MANGATAL L, ADELINE MT, GUÉNARD D, GUÉRITTE-VOEGELEIN F AND POTIER P. (1989). Application of the vicinal hydroxyamination reaction with asymmetric induction to the hemisynthesis of taxol and analogs. *Tetrahedron*, **45**, 4177-4190.

NA GC AND TIMASHEFF SN. (1982). *In vitro* vinblastine-induced tubulin paracrystals. *J. Biol. Chem.*, **257**, 10387-10391.

PARNESS J AND HORWITZ SB. (1981). Taxol binds to polymerized tubulin *in vitro*. *J. Cell Biol.*, **91**, 479-487.

PRAKASH V AND TIMASHEFF SN. (1983). The interaction of vincristine with calf brain tubulin. *J. Biol. Chem.*, **258**, 1689-1697.

RINGEL I AND HORWITZ SB. (1991). Studies with RP 56976 (Taxotere): a semisynthetic analogue of Taxol. *J. Natl Cancer Inst.*, **83**, 288-291.

RIOU J-F, NAUDIN A AND LAVELLE F. (1992). Effects of Taxotere on murine and human tumour cell lines. *Biochem. Biophys. Res. Commun.*, **187**, 164-170.

RIOU J-F, PETITGENET O, COMBEAU C AND LAVELLE F. (1994). Cellular uptake and efflux of docetaxel (Taxotere) and paclitaxel (Taxol) in P388 cell line. *Proc. Am. Assoc. Cancer Res.*, **35**, 385.

ROBBINS E, JENTZSCH G AND MICALI A. (1968). The centriole cycle in synchronized HeLa cells. *J. Cell Biol.*, **36**, 329-339.

ROBERTS JR, ROWINSKY EK, DONEHOWER RC, ROBERTSON J AND ALLISON DC. (1989). Demonstration of the cell cycle positions of taxol-induced asters and bundles by sequential measurements of tubulin immunofluorescence, DNA content, and autoradiographic labeling of taxol-sensitive and -resistant cells. *J. Histochem. Cytochem.*, **37**, 1659-1665.

ROBERTS JR, ALLISON DC, DONEHOWER RC AND ROWINSKY EK. (1990). Development of polyploidization in taxol-resistant human leukemia cells *in vitro*. *Cancer Res.*, **50**, 710-716.

ROWINSKY EK, DONEHOWER RC, JONES RJ AND TUCKER RW. (1988). Microtubule changes and cytotoxicity in leukemic cell lines treated with taxol. *Cancer Res.*, **48**, 4093-4100.

ROWINSKY EK, ONETTO N, CANETTA RM AND ARBUCK SG. (1992). Taxol: the first of the taxanes, an important new class of antitumor agents. *Semin. Oncol.*, **19**, 646-662.

SCHIFF PB AND HORWITZ SB. (1980). Taxol stabilizes microtubules in mouse fibroblast cells. *Proc. Natl Acad. Sci. USA*, **77**, 1561-1565.

SCHIFF PB AND HORWITZ SB. (1981). Taxol assembles tubulin in the absence of exogenous guanosine 5'-triphosphate or microtubule-associated proteins. *Biochemistry*, **20**, 3247-3252.

SCHIFF PB, FANT J AND HORWITZ SB. (1979). Promotion of microtubule assembly *in vitro* by taxol. *Nature*, **277**, 665-667.

SKODA RC, JAUSSI R AND CHRISTEN P. (1983). Vinblastine inhibits the maturation of the precursor of mitochondrial aspartate aminotransferase. Vincristine and six other cytoskeleton inhibitors do not show this effect. *Biochem. Biophys. Res. Commun.*, **115**, 144-152.

THOMPSON WC, WILSON L AND PURICH DL. (1981). Taxol induces microtubule assembly at low temperature. *Cell Motil.*, **1**, 445-454.

TOURNIER F AND BORNENS M. (1994). Cell cycle regulation of centrosome function. In *Microtubules*, Hyams JS and Lloyd CW. (eds) pp. 303-324. John Wiley: New York.

TSAO Y-P, D'ARPA P AND LIU LF. (1992). The involvement of active DNA synthesis in camptothecin-induced G2 arrest: altered regulation of P34cdc2 cyclin B. *Cancer Res.*, **52**, 1823-1829.

WANI MC, TAYLOR HL, WALL ME, COGGON P AND McPHAIL AT. (1971). Plant antitumor agents. VI. Isolation and structure of taxol, a novel antileukemic and antitumor agent from *Taxus Brevifolia*. *J. Am. Chem. Soc.*, **93**, 2325-2327.